# Supplementary material for: When can AlphaFold predict the oligomeric states of proteins?
Source: Protein Sci. 2026 Jun 16;35(7):e70686. doi: 10.1002/pro.70686 (PMC13270398; doi:10.1002/pro.70686)
Supplement: Supplementary file 1 — TABLE S1. Protein set. TABLE S2. Comparison of oligomer predictions to experimentally resolved structures and AF2 monomer. TABLE S3. Membrane protein set. FIGURE S1. ipTM range and pLDDT plots for AF2‐M predictions. FIGURE S2. Assignment of oligomeric state using ipSAE for the initial test set of 40 proteins. FIGURE S3. Bootstrapping analysis for the TM set of 1006 proteins. FIGURE S4. Proportion of and proteins predicted in the correct oligomeric state by AF2‐M for proteins with Foldseek TM score <0.5 and >0.5. FIGURE S5. Proportion of proteins predicted in the correct oligomeric state by AF2‐M using different confidence metrics for proteins with Foldseek TM score <0.5 and >0.5. FIGURE S6. Diversity of organisms and success rates against sequence length, monomer pLDDT, Foldseek TM Score, and MMseqs2 Score for the set of 3560 proteins. FIGURE S7. ipTM distribution plots for AF3 predictions. FIGURE S8. Structural images of AF3 predictions. [file PRO-35-e70686-s001.docx]

**Supporting Information**

**Table S1 – Protein Set**

| UniProt ID | Annotated Oligomeric State | PDB Available? | PDB in Training Set? | Monomer pLDDT | Correct using AF2.3? | Correct using AF3? | Membrane Localisation | Membrane Protein Topology | Max Foldseek TM Score | X-Group(s) |
| --- | --- | --- | --- | --- | --- | --- | --- | --- | --- | --- |
| Q13087 | 1 | ✘ | ✘ | 87.53 | ✔ | ✘ | ✘ | N/A | 0.94 | Thioredoxin-like |
| Q9NZ08 | 1 | ✔ | ✔ | 92.76 | ✔ | ✘ | ✔ | Single-pass | 1.00 | Repetitive alpha hairpins,  Baculovirus p35 protein-related    Immunoglobulin-like beta-sandwich  Zincin-like |
| P30740 | 1 | ✔ | ✔ | 92.93 | ✔ | ✔ | ✘ | N/A | 0.99 | Serpins |
| P27144 | 1 | ✔ | ✔ | 90.22 | ✔ | ✔ | ✘ | N/A | 0.97 | Rubredoxin-like, P-loop domains-like |
| Q68CK6 | 1 | ✘ | ✘ | 91.42 | ✔ | ✔ | ✘ | N/A | 1.00 | Alpha-lytic protease prodomain-like, CoA-dependent acyltransferases |
| Q16762 | 1 | ✔ | ✘ | 96.43 | ✔ | ✔ | ✘ | N/A | 1.00 | Flavodoxin-like |
| P35670 | 1 | ✔ | ✔ | 71.83 | ✔ | ✘ | ✔ | Multi-pass | 1.00 | Alpha-beta plaits, Calcium ATPase transmembrane domain-related, Metal cation-transporting ATPase, ATP-binding domain, jelly-roll, HAD domain-like |
| Q68D85 | 1 | ✔ | ✔ | 78.19 | ✔ | ✔ | ✔ | Single-pass | 0.99 | Immunoglobulin-like beta-sandwich, Retroviral matrix proteins |
| Q96QG7 | 2 | ✘ | ✘ | 94.9 | ✔ | ✔ | ✔ | N/A | 0.95 | Flavodoxin-like  , PH domain-like |
| P06727 | 2 | ✔ | ✔ | 80.45 | ✘ | ✔ | ✘ | N/A | 0.90 | Four-helical up-and-down bundle |
| O94804 | 2 | ✔ | ✔ | 74.81 | ✘ | ✔ | ✔ | N/A | 0.98 | Protein kinase/SAICAR synthase/ATP-grasp |
| Q9P0L0 | 2 | ✔ | ✔ | 79.7 | ✔ | ✔ | ✔ | Single-pass | 1.00 | Immunoglobulin-like beta-sandwich |
| O43776 | 2 | ✔ | ✔ | 94.06 | ✔ | ✔ | ✘ | N/A | 0.93 | Asparaginal-tRNA synthetase N-terminal domain,  Class II aaRS and biotin synthetases  OB-fold    Class II aaRS and biotin synthetases |
| P20711 | 2 | ✔ | ✔ | 96.94 | ✔ | ✔ | ✘ | N/A | 0.71 | PLP-dependent transferases    C-terminal domain in some PLP-dependent transferases |
| Q9GZP0 | 2 | ✘ | ✘ | 74.79 | ✔ | ✘ | ✘ | N/A | 0.94 | Cystine-knot cytokines  jelly-roll |
| Q2M3C6 | 2 | ✘ | ✘ | 54.62 | ✘ | ✘ | ✔ | Multi-pass | 0.98 | Voltage-gated ion channels |
| P32970 | 3 | ✔ | ✔ | 83.43 | ✔ | ✔ | ✔ | Single-pass | 0.94 | jelly-roll |
| P43005 | 3 | ✔ | ✔ | 80.33 | ✔ | ✔ | ✔ | Multi-pass | 0.98 | Proton glutamate symport protein |
| Q8TCS8 | 3 | ✔ | ✔ | 87.53 | ✔ | ✔ | ✔ | N/A | 0.99 | HTH  Ribonuclease PH domain 2-like    Ribosomal protein S5 domain 2-like    6, Alpha-lytic protease prodomain-like |
| P21941 | 3 | ✘ | ✘ | 83.72 | ✔ | ✘ | ✘ | N/A | 0.99 | HAD domain-like  EGF-like |
| P50591 | 3 | ✔ | ✔ | 82.4 | ✔ | ✔ | ✔ | Single-pass | 1.00 | jelly-roll  iron-sulfur subunit (ISP) transmembrane anchor |
| O14684 | 3 | ✔ | ✔ | 96.25 | ✔ | ✔ | ✔ | Multi-pass | 1.00 | Cytochrome c oxidase subunit I-like |
| P43003 | 3 | ✔ | ✔ | 81.85 | ✔ | ✔ | ✔ | Multi-pass | 0.98 | Proton glutamate symport protein |
| Q99735 | 3 | ✔ | ✔ | 93.94 | ✔ | ✔ | ✔ | Multi-pass | 1.00 | Cytochrome c oxidase subunit I-like |
| Q9NPJ3 | 4 | ✔ | ✔ | 96.16 | ✔ | ✔ | ✘ | N/A | 1.00 | Thioesterase/thiol ester dehydrase-isomerase-like |
| O75496 | 4 | ✔ | ✔ | 70.4 | ✘ | ✔ | ✘ | N/A | 1.00 | - |
| O60741 | 4 | ✔ | ✔ | 70.56 | ✔ | ✔ | ✔ | Multi-pass | 0.99 | jelly-roll  Voltage-gated ion channels |
| O43827 | 4 | ✘ | ✘ | 86.23 | ✘ | ✘ | ✘ | N/A | 0.96 | Fibrinogen C-terminal domain-like |
| Q8N539 | 4 | ✔ | ✔ | 80.24 | ✔ | ✔ | ✔ | Single-pass | 1.00 | Fibrinogen C-terminal domain-like  Mediator hinge subcomplex-like |
| P02766 | 4 | ✔ | ✔ | 88.93 | ✔ | ✔ | ✘ | N/A | 1.00 | Immunoglobulin-like beta-sandwich |
| Q9P0L9 | 4 | ✔ | ✔ | 76.04 | ✔ | ✔ | ✔ | Multi-pass | 0.98 | Polycystin-mucolipin domain  Voltage-gated ion channels  EF-hand |
| P53396 | 4 | ✔ | ✔ | 92.6 | ✔ | ✔ | ✘ | N/A | 1.00 | Flavodoxin-like  Protein kinase/SAICAR synthase/ATP-grasp  Rossmann-like |
| P23415 | 5 | ✔ | ✔ | 84.63 | ✔ | ✔ | ✔ | Multi-pass | 0.99 | Neurotransmitter-gated ion-channel transmembrane pore  Immunoglobulin-like beta-sandwich |
| P02743 | 5 | ✔ | ✔ | 94.41 | ✔ | ✔ | ✘ | N/A | 1.00 | jelly-roll |
| Q68DU8 | 5 | ✔ | ✔ | 69.39 | ✔ | ✔ | ✔ | N/A | 1.00 | POZ domain |
| Q9NXV2 | 5 | ✔ | ✔ | 78.28 | ✔ | ✔ | ✘ | N/A | 1.00 | POZ domain |
| P02741 | 5 | ✔ | ✔ | 94.23 | ✔ | ✔ | ✘ | N/A | 1.00 | jelly-roll |
| O75311 | 5 | ✔ | ✔ | 84.36 | ✔ | ✔ | ✔ | Multi-pass | 0.99 | Neurotransmitter-gated ion-channel transmembrane pore |
| Q693B1 | 5 | ✘ | ✘ | 84.28 | ✔ | ✔ | ✘ | N/A | 0.89 | Immunoglobulin-like beta-sandwich |
| Q86SE8 | 5 | ✔ | ✔ | 73.32 | ✔ | ✔ | ✘ | N/A | 1.00 | jelly-roll |

**Table S2 – Comparison of oligomer predictions to experimentally resolved structures and AF2 monomer**

| **UniProt** | **# PDB** | **# Eligible PDB (seq similarity & length)** | **Eligible PDBs** | **Max TM Score (AF2)** | **Most similar PDB (AF2)** | **TM Score to Monomer (AF2)** | **Max TM Score (AF3)** | **Most similar PDB (AF3)** | **TM Score to Monomer (AF3)** |
| --- | --- | --- | --- | --- | --- | --- | --- | --- | --- |
| Q96QG7 | 0 | 0 |  | - | - | 0.965 | - | - | 0.957 |
| P06727 | 1 | 1 | 3S84 | 0.375 | 3S84 | 0.249 | 0.954 | 3S84 | 0.566 |
| O94804 | 14 | 0 |  | - | - | 0.596 | - | - | 0.637 |
| Q9P0L0 | 2 | 2 | 2RR3, 6TQR | 0.973 | 6TQR | 0.558 | 0.974 | 6TQR | 0.571 |
| O43776 | 6 | 5 | 5XIX, 8H53, 8TC7, 8TC8, 8TC9 | 0.996 | 8TC9 | 0.916 | 0.995 | 8TC7 | 0.971 |
| P20711 | 5 | 3 | 3RBF, 3RBL, 3RCH | 0.869 | 3RCH | 0.978 | 0.877 | 3RCH | 0.995 |
| Q9GZP0 | 0 | 0 |  | - | - | 0.390 | - | - | 0.422 |
| Q2M3C6 | 0 | 0 |  | - | - | 0.316 | - | - | 0.323 |
| P32970 | 1 | 1 | 7KX0 | 0.968 | 7KX0 | 0.715 | 0.994 | 7KX0 | 0.731 |
| P43005 | 14 | 9 | 6S3Q, 6X2L, 6X3E, 6X3F, 7NSG, 8CTC, 8CUA, 8CUI, 8CV2 | 0.988 | 8CV2 | 0.884 | 0.998 | 6X2L | 0.584 |
| Q8TCS8 | 2 | 1 | 3U1K | 0.989 | 3U1K | 0.885 | 0.987 | 3U1K | 0.885 |
| P21941 | 0 | 0 |  | - | - | 0.399 | - | - | 0.422 |
| P50591 | 7 | 4 | 1D0G, 1DU3, 4N90, 5CIR | 0.969 | 3U1K | 0.562 | 0.990 | 5CIR | 0.583 |
| O14684 | 18 | 1 | 3DWW | 0.825 | 3DWW | 0.978 | 0.823 | 3DWW | 0.976 |
| P43003 | 5 | 1 | 7NPW | 0.776 | 7NPW | 0.907 | 0.772 | 7NPW | 0.868 |
| Q99735 | 4 | 4 | 6SSR, 6SSS, 6SSU, 6SSW | 0.989 | 6SSS | 0.910 | 0.992 | 6SSS | 0.916 |
| Q9NPJ3 | 3 | 2 | 2F0X, 3F5O | 0.996 | 2F0X | 0.994 | 0.997 | 2F0X | 0.993 |
| O75496 | 6 | 0 |  | - | - | 0.382 | - | - | 0.334 |
| O60741 | 11 | 11 | 5U6O, 5U6P, 6UQF, 6UQG, 8T4M, 8T4Y, 8T50, 8UC7, 8UC8, 9BC6, 9BC7 | 0.979 | 8T4Y | 0.616 | 0.997 | 8T4M | 0.655 |
| O43827 | 0 | 0 |  | - | - | 0.632 | - | - | 0.753 |
| Q8N539 | 8 | 0 |  | - | - | 0.507 | - | - | 0.496 |
| P02766 | 412 | 41 | 1G1O, 1GKO, 1ICT, 1QAB, 1RLB, 1SOQ, 1TZ8, 2QEL, 2WQA, 3BSZ, 3DGD, 3DID, 3DO4, 3GPS, 3GRB, 3GRG, 5EZP, 5LLV, 5TTR, 6SDZ, 7OB4, 7Y1I, 8ADE, 8E7D, 8E7E, 8E7H, 8E7I, 8E7J, 8G9R, 8GBR, 8I0O, 8PKE, 8PKF, 8PKG, 8TDN, 8TDO, 8VE0, 8VE1, 8VE2, 8VE3, 8VE4 | 0.992 | 8VE1 | 0.821 | 0.996 | 8VE1 | 0.818 |
| Q9P0L9 | 3 | 1 | 6DU8 | 0.981 | 6DU8 | 0.759 | 0.992 | 6DU8 | 0.625 |
| P53396 | 35 | 20 | 6HXH, 6O0H, 6POE, 6POF, 6QFB, 6UI9, 6UIA, 6UUW, 6UUZ, 6UV5, 7LIW, 7LJ9, 7LLA, 7RIG, 7RKZ, 7RMP, 8G1E, 8G1F, 8G5C, 8G5D | 0.974 | 8G5C | 0.915 | 0.999 | 6UUW | 0.961 |
| P23415 | 9 | 3 | 8DN2, 8DN4, 8DN5 | 0.990 | 8DN4 | 0.834 | 0.992 | 8DN4 | 0.830 |
| P02743 | 13 | 13 | 1GYK, 1LGN, 1SAC, 2A3W, 2A3X, 2A3Y, 2W08, 3D5O, 3KQR, 4AVS, 4AVT, 4AVV, 4AYU | 0.997 | 2W08 | 0.919 | 0.999 | 4AVV | 0.934 |
| Q68DU8 | 7 | 0 |  | - | - | 0.320 | - | - | 0.299 |
| Q9NXV2 | 9 | 2 | 3DRX, 3DRY | 0.914 | 3DRX | 0.719 | 0.942 | 3DRX | 0.720 |
| P02741 | 16 | 16 | 1B09, 1GNH, 1LJ7, 3L2Y, 3PVN, 3PVO, 7PK9, 7PKB, 7PKD, 7PKE, 7PKF, 7PKG, 7PKH, 7TBA, 8WV4, 8WV5 | 0.997 | 7PKB | 0.945 | 0.999 | 7PKE | 0.945 |
| O75311 | 5 | 5 | 5CFB, 5TIN, 5TIO, 5VDH, 5VDI | 0.991 | 5TIO | 0.815 | 0.996 | 5TIO | 0.799 |
| Q693B1 | 0 | 0 |  | - | - | 0.683 | - | - | 0.663 |
| Q86SE8 | 1 | 1 | 3T30 | 0.995 | 3T30 | 0.570 | 0.992 | 3T30 | 0.603 |

**Table S3 – Membrane Protein Set**

| UniProt ID | Annotated Oligomeric State | PDB Available? | PDB in Training Set? | Monomer pLDDT | Correct using AF2.3? | Membrane Localisation? | Membrane Protein Topology | Max Foldseek TM Score | X-Group |
| --- | --- | --- | --- | --- | --- | --- | --- | --- | --- |
| O94886 | 1 | ✔ | ✘ | 75.3 | ✘ | ✔ | Multi-pass | 0.86 | TMEM16 lipid scramblase transmembrane domain |
| P31645 | 1 | ✔ | ✔ | 85.43 | ✘ | ✔ | Multi-pass | 0.99 | Sodium:neurotransmitter symporter family (SNF)-like |
| Q9UBH6 | 2 | ✔ | ✔ | 83.73 | ✔ | ✔ | Multi-pass | 0.90 | SPX domain  Family A G protein-coupled receptor-like |
| Q9XEA1 | 2 | ✔ | ✔ | 84.81 | ✔ | ✔ | Multi-pass | 0.99 | Alpha-beta plaits    TMEM16 lipid scramblase transmembrane domain  Repetitive alpha hairpins |
| P43004 | 3 | ✔ | ✘ | 77.74 | ✔ | ✔ | Multi-pass | 0.97 | Proton glutamate symport protein |
| Q1XA76 | 3 | ✔ | ✔ | 83.42 | ✔ | ✔ | Multi-pass | 0.98 | Amiloride-sensitive cation channel 2 |
| A8EVM5 | 4 | ✔ | ✔ | 93.73 | ✔ | ✔ | Multi-pass | 1.00 | Voltage-gated ion channels |
| Q8NET8 | 4 | ✔ | ✔ | 76.93 | ✔ | ✔ | Multi-pass | 0.98 | Repetitive alpha hairpins    Voltage-gated ion channels |
| O77389 | 5 | ✔ | ✔ | 91.42 | ✔ | ✔ | Multi-pass | 0.95 | Aquaporin-like |
| P0A742 | 5 | ✔ | ✔ | 85.22 | ✔ | ✔ | Multi-pass | 0.57 | Gated mechanosensitive channel |
| P23416 | 5 | ✔ | ✔ | 84.44 | ✔ | ✔ | Multi-pass | 0.99 | Neurotransmitter-gated ion-channel transmembrane pore  Immunoglobulin-like beta-sandwich |
| Q09068 | 6 | ✘ | ✘ | 90.27 | ✔ | ✔ | Multi-pass | 0.96 | Acid-activated urea channel |
| P0C0S1 | 7 | ✔ | ✔ | 92.62 | ✔ | ✔ | Multi-pass | 0.95 | Mechanosensitive channel protein MscS (YggB), transmembrane region  Alpha-beta plaits  SH3 |
| Q96RD6 | 7 | ✔ | ✘ | 58.15 | ✔ | ✔ | Multi-pass | 0.90 | Connexin 26 |

**Table S4 – Extended Membrane Protein Set**

**See supplementary File**

**Table S5 – TM Set of 1006 Proteins**

**See supplementary File**

**Table S6 – X Group Set of 3560 Proteins**

**See supplementary File**

**Table S7 – Overall accuracy across X Groups**

**See supplementary file**

**
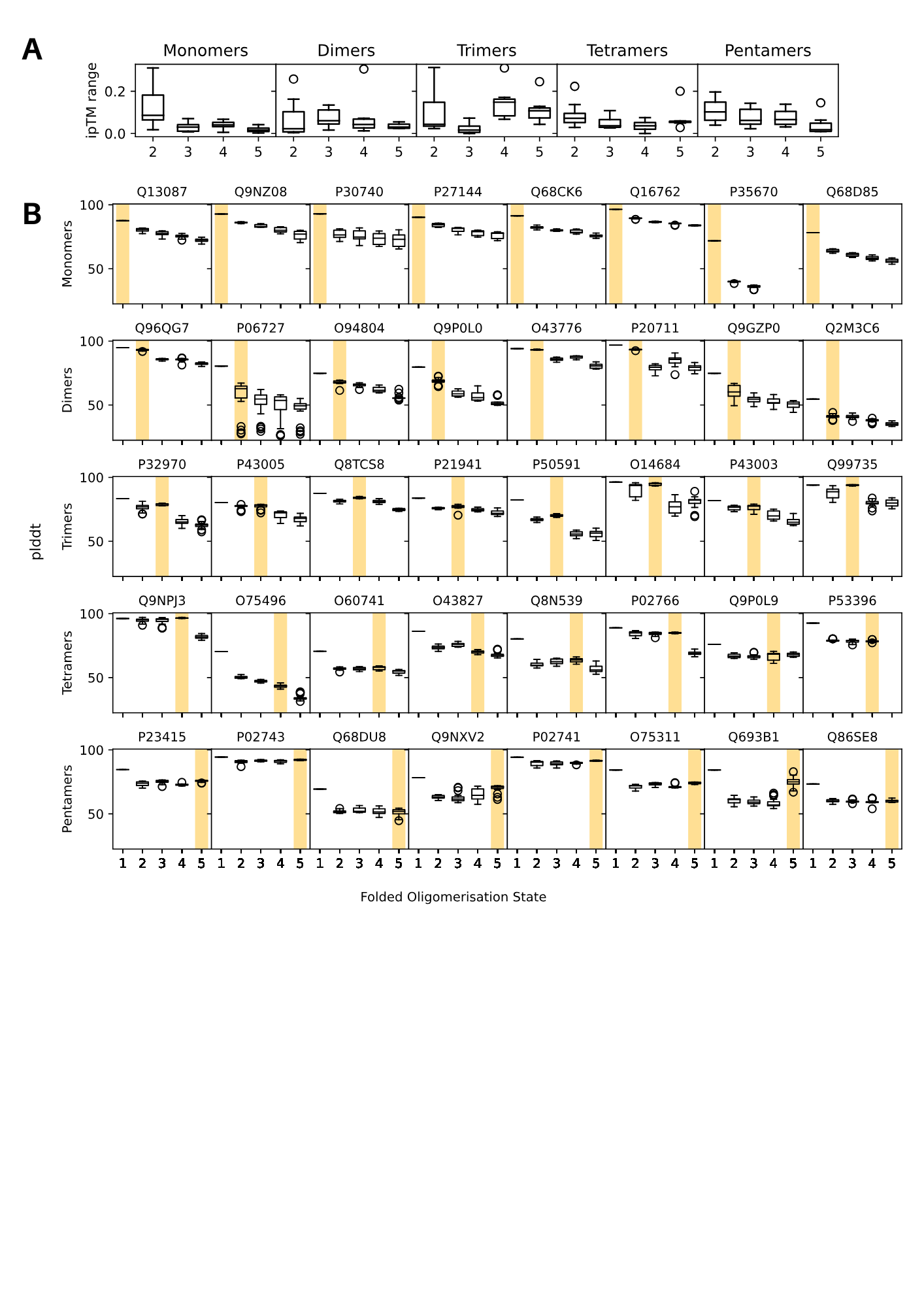
**

**Figure S1 – ipTM range and pLDDT plots for AF2-M predictions**

1. ipTM range distributions for monomeric, dimeric, trimeric, tetrameric and pentameric proteins in different folded oligomeric states
2. Box plots showing pLDDT score distributions for each protein in different folded oligomeric states across the 40 test set proteins. The annotated oligomeric state highlighted in yellow.


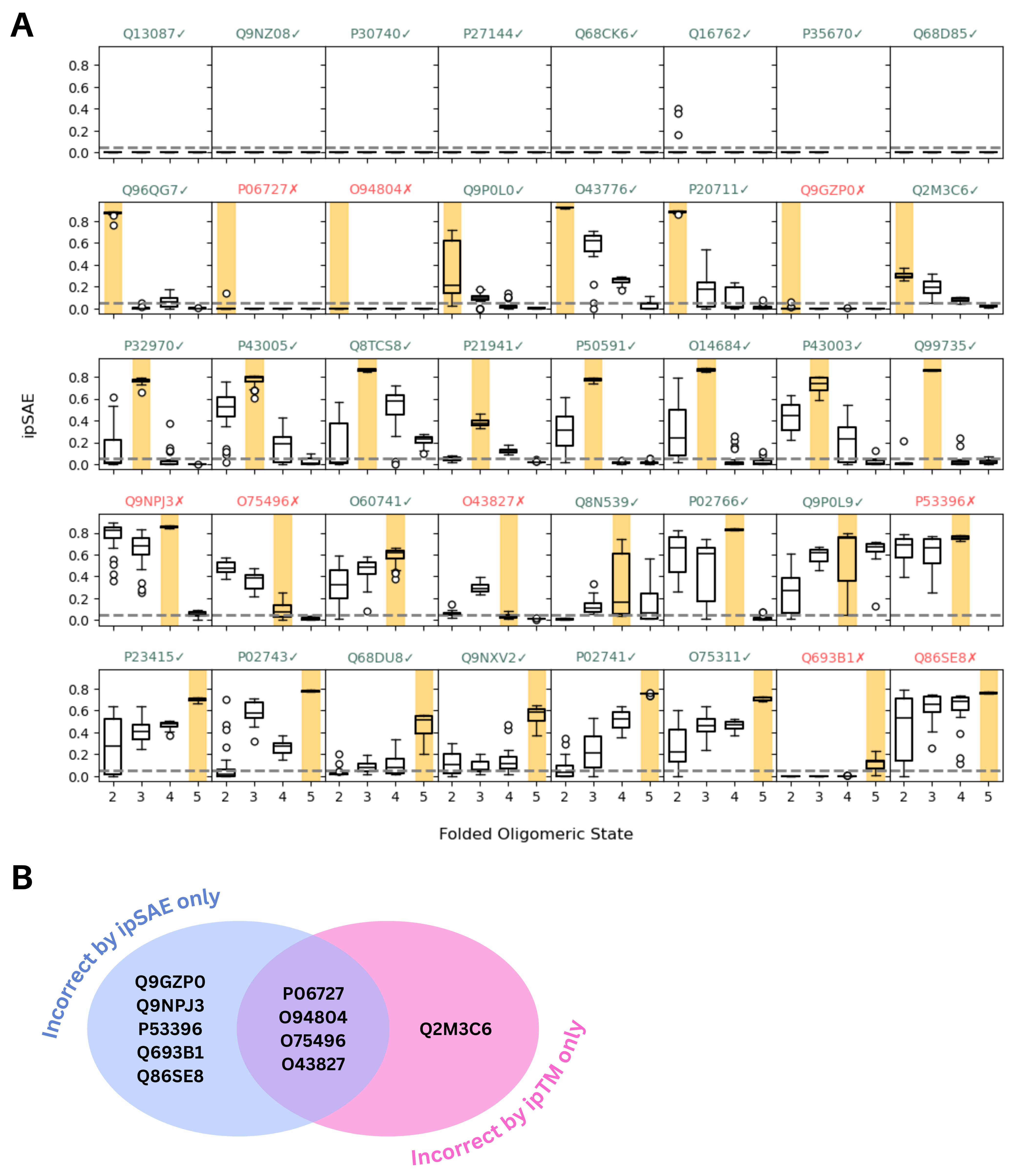


**Figure S2 - Assignment of oligomeric state using ipSAE for the initial test set of 40 proteins.**

1. ipSAE range distributions for monomeric, dimeric, trimeric, tetrameric and pentameric proteins in different folded oligomeric states.
2. Venn diagram of proteins assigned incorrectly using ipTM (pink) and ipSAE (blue).


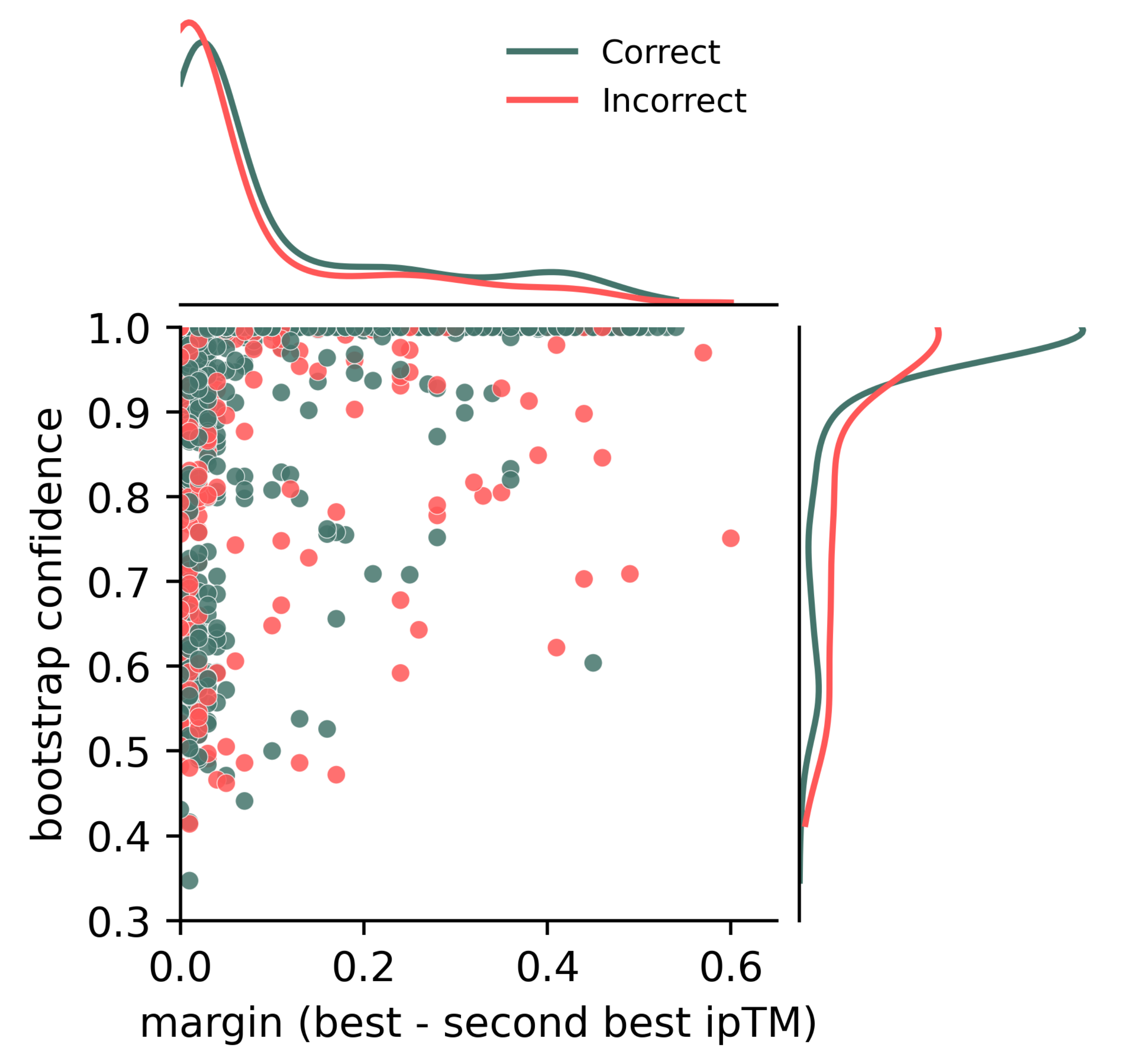


**Figure S3 – Bootstrapping analysis for the TM set of 1006 proteins.**

**The bootstrap confidence (proportion of time the same oligomeric state predicted from all 20 structures is assigned based on a random sample of one structure per oligomeric state out of 1000 trials) and margin (difference in max ipTM between between the top and second ranked oligomeric state) for proteins in the TM set of 1006 proteins, coloured by their the accuracy of the oligomeric state assignment**


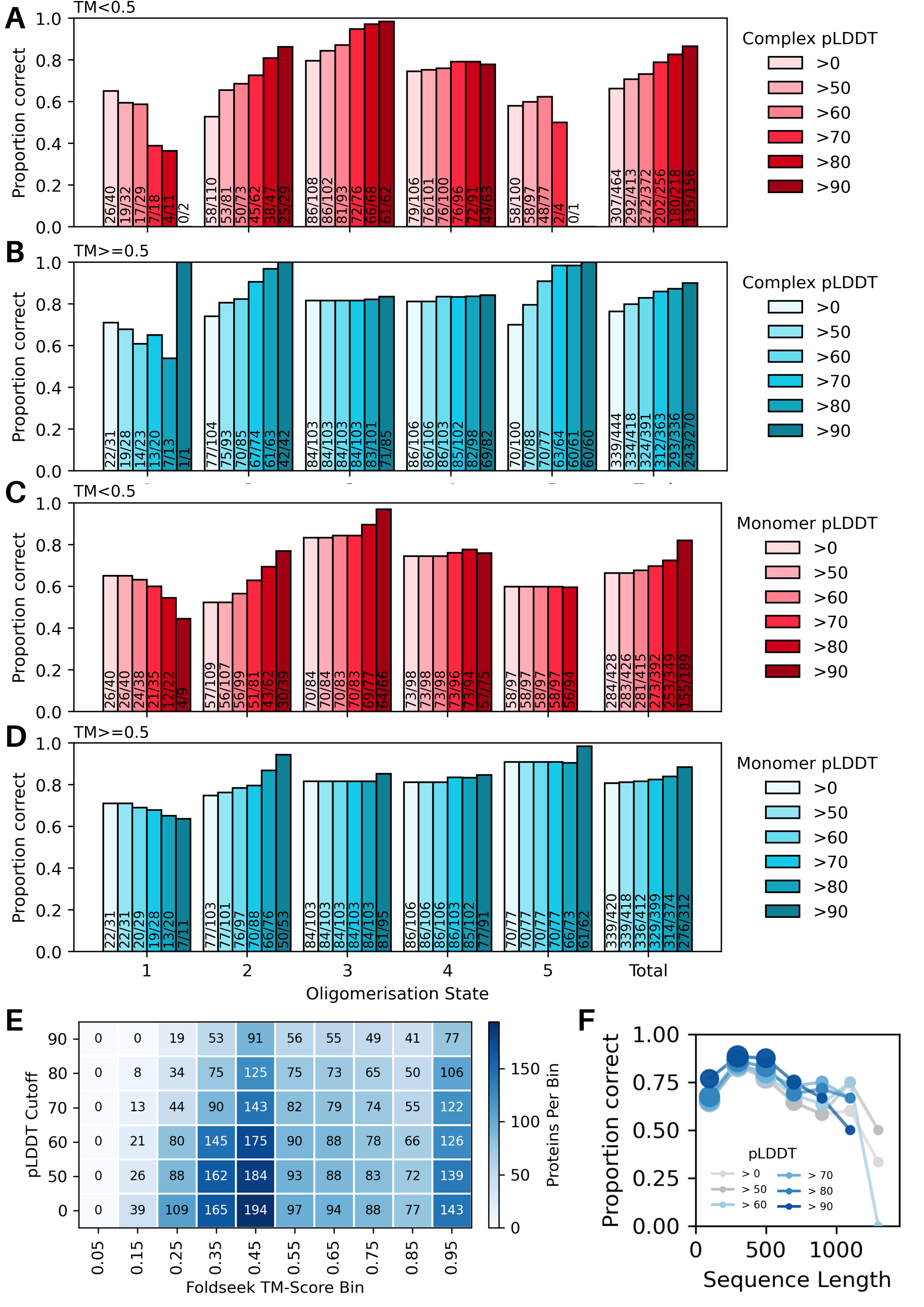


**Figure S4 - Proportion of and proteins predicted in the correct oligomeric state by AF2-M for proteins with Foldseek TM score < 0.5 and > 0.5.**

1. (A-B) Proportion of proteins predicted correctly by AF2-M from (A) a set of approximately 500 with Foldseek TM score < 0.5 to any protein in the AlphaFold training set; and (B) approximately 500 with Foldseek TM score > 0.5 to at least one protein in the AlphaFold training set, filtered by different pLDDT cutoffs for the predicted oligomeric complexes. For monomeric proteins, the pLDDT value used corresponds to the folded oligomeric state with the highest mean ipTM. Breakdowns are shown for the different folded oligomeric states as well as the overal total number of correct proteins. The number of correct proteins out of the total number of proteins in each bar is displayed in the graph.

(C-D) Proportion of proteins predicted correctly by AF2-M from (C) a set of approximately 500 with Foldseek TM score < 0.5 to any protein in the AlphaFold training set; and (D) approximately 500 proteins with Foldseek TM score > 0.5 to at least one protein in the AlphaFold training set, filtered by different pLDDT cutoffs for the AFDB monomeric structure. Breakdowns are shown for the different folded oligomeric states as well as the overal total number of correct proteins. The number of correct proteins out of the total number of proteins in each bar is displayed in the graph.

(E) Number of proteins per pLDDT cutoff per Foldseek TM-Score Bin for data shown in Fig. 4B.

(F) Proportion of proteins predicted correctly versus the length of the sequence. Data are filtered by pLDDT of the predicted oligomeric complex (or of the AFDB monomer structure, in the case of monomeric proteins).


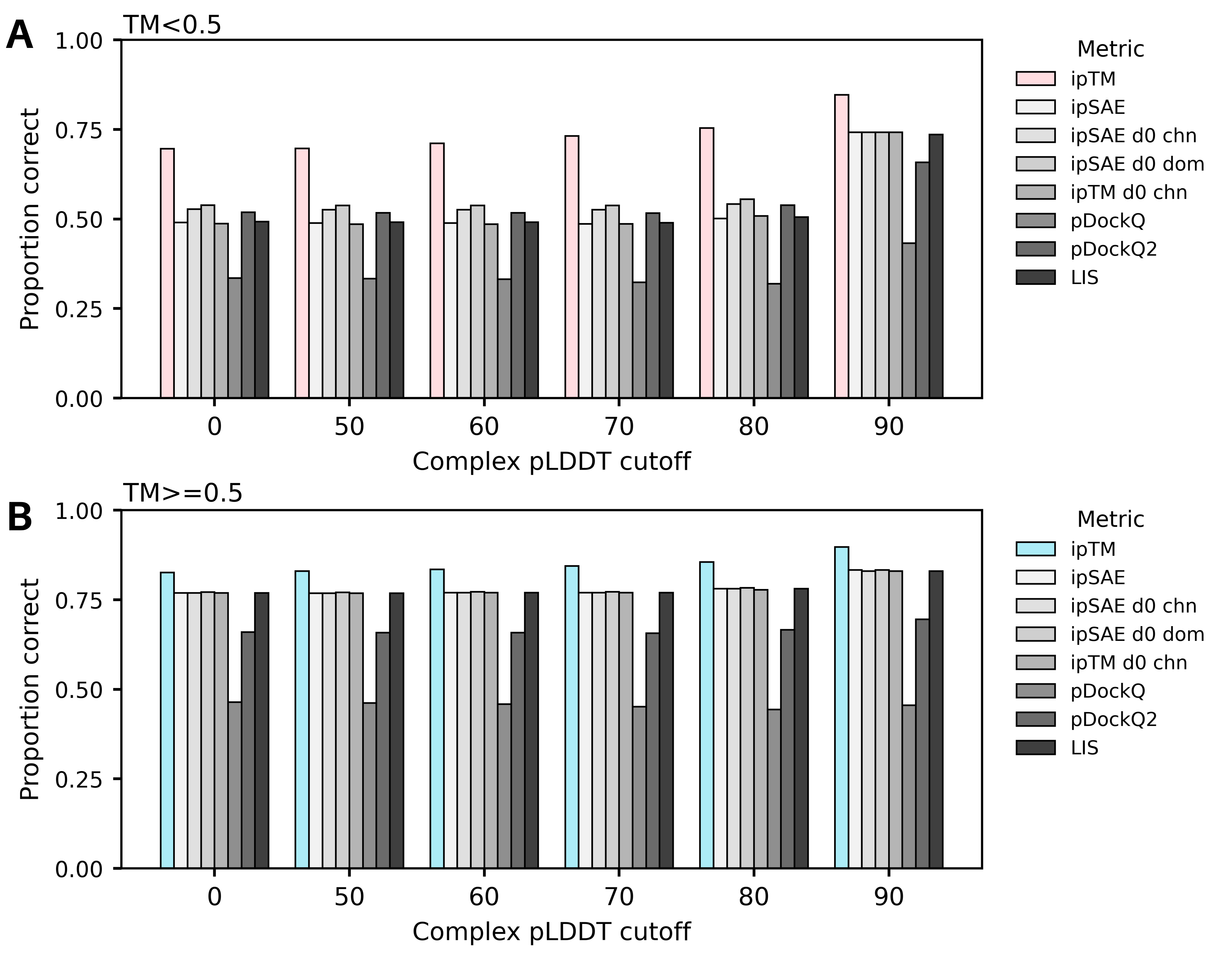


**Figure S5 – Proportion of proteins predicted in the correct oligomeric state by AF2-M using different confidence metrics for proteins with Foldseek TM score < 0.5 and > 0.5.**

(A-B) Proportion of proteins with (A) Foldseek TM scores < 0.5 or (B) > 0.5 to any protein in the AlphaFold training set that were predicted correctly using different interface confidence metrics, including ipSAE, ipSAE d0 chn, ipSAE d0 dom, ipTM d0 chn, pDockQ, pDockQ2, LIS, and ipTM. Performance was assessed after filtering out structures based on pLDDT.


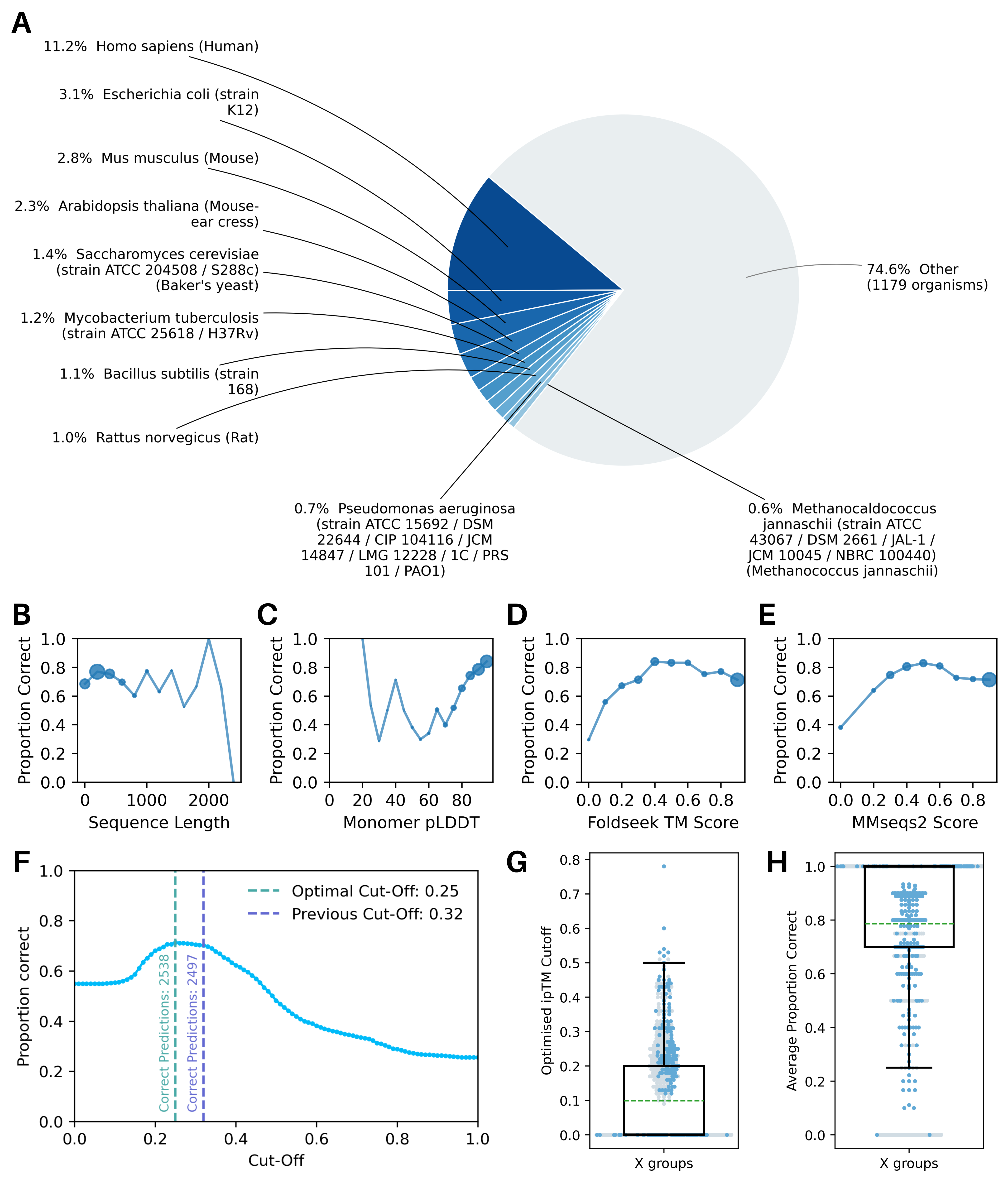


**Figure S6 – Diversity of organisms and success rates against sequence length, monomer pLDDT, Foldseek TM Score, and MMseqs2 Score for the set of 3,560 proteins.**

1. Pie chart of organisms in the X Group Set. The top 10 most abundant organisms are shown in blue wedges. The remaining 1179 organisms are grouped in the grey wedge.

(B-E) Proportion correct versus (B) Sequence Length, (C) Monomer pLDDT, (D) Foldseek TM Score, (E) MMseqs2 Score. Marker size is proportional to the number of proteins per bin.

(F) Optimising the ipTM cutoff for the X Group Set of 3,560 proteins. IpTM cutoff was optimised using the Mean of Max method described previously. The optimal cut-off was determined to be 0.25.

(G-H) Optimising the ipTM cutoff for each X Group. The ipTM cutoff that assigns the most proteins to their annotated oligomeric state was determined for each X Group. Boxplots show the (G) optimised ipTM cutoffs for each X Group and (H) average proportion of proteins assigned correctly for each X Group.


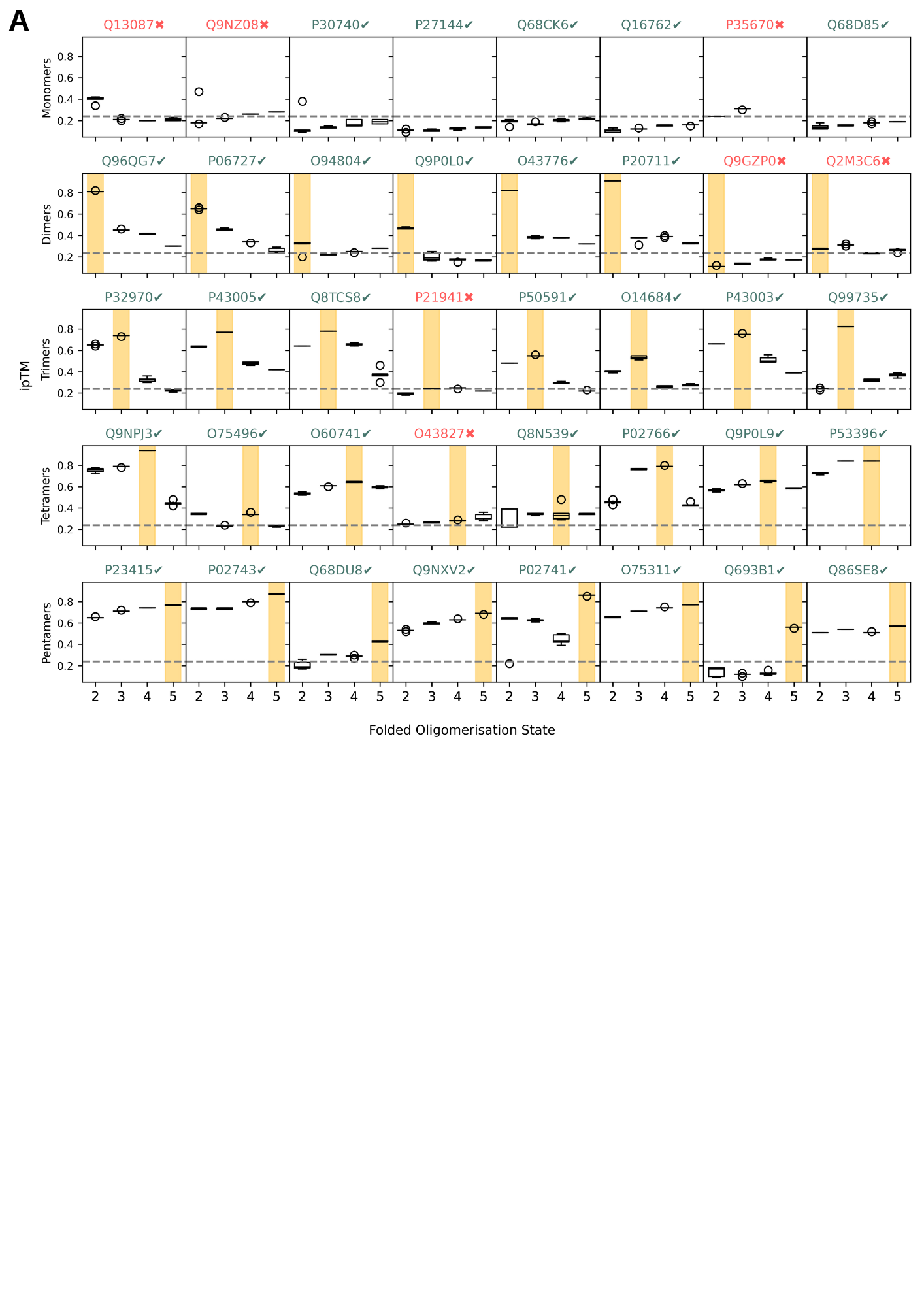
**Figure S7 – ipTM distribution plots for AF3 predictions**

1. Box plots showing ipTM score distributions for each protein in different folded oligomeric states across the 40 test set proteins. The annotated oligomeric state highlighted in yellow. UniProt IDs are noted in green for correct predictions and red for incorrect ones.

**
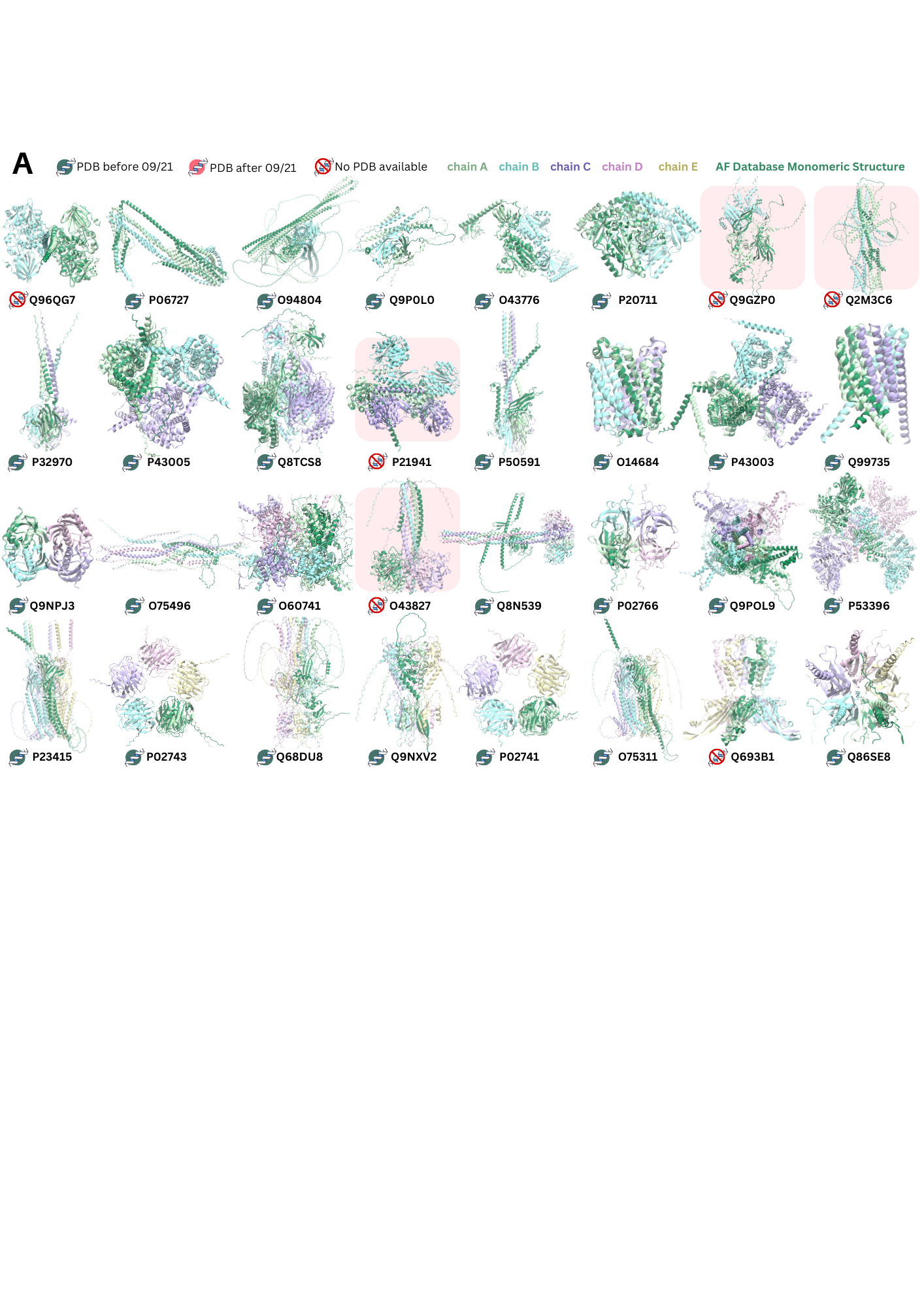
**

**Figure S8 – structural images of AF3 predictions**

1. Snapshots of the top ranked structural predictions by AF3 in the correct oligomeric state for each multimeric protein (ribbon, coloured by chain), aligned with the AFDB prediction of the protein as a monomer (ribbon, dark green). The presence of an experimentally resolved structure in the PDB or AlphaFold3 training set is indicated with an icon near each UniProt ID. The structures of proteins predicted in an incorrect oligomeric state are highlighted in red.
